# Supplementary material for: Practical Management of Biosimilar Use in Inflammatory Bowel Disease (IBD): A Global Survey and an International Delphi Consensus
Source: J Clin Med. 2023 Oct 3;12(19):6350. doi: 10.3390/jcm12196350 (PMC10574001; doi:10.3390/jcm12196350)
Supplement: Supplementary file 1 [file jcm-12-06350-s001.zip › jcm-2602895-supplementary.pdf]

Supplementary Table 1. Integral survey with responses

SURVEY ON THE USE OF BIOSIMILARS		
Q1. Age in years:		
**Answered**	**234**	
**Skipped**	**0**	
Q2. Sex		
Answer Choices	Responses	
Male	56.41%	132
Female	43.59%	102
	**Answered**	**234**
	**Skipped**	**0**
Q3. What country do you work in?		
**Answered**	**234**	
**Skipped**	**0**	
Q4. What is your specialization?		
Answer Choices	Responses	
Gastroenterologist	86.32%	202
Internal doctor	1.28%	3
Surgeon	2.56%	6
General practitioner	1.28%	3
Other (please specify)	8.55%	20
	**Answered**	**234**
	**Skipped**	**0**
Q5. How many years of experience do you have in the field of IBD?		
Answer Choices	Responses	
Less than 1 year	0.43%	1
Less than 5 years	6.84%	16
Less than 10 years	18.38%	43
More than 10 years	74.36%	174
	**Answered**	**234**
	**Skipped**	**0**
Q6. How many IBD patients do you see per year?		
Answer Choices	Responses	
Less than 100	24.36%	57
Less than 500	38.46%	90
Less than 1000	23.93%	56

More than 1000	13.25%	31
	**Answered**	**234**
	**Skipped**	**0**
Q7. In your opinion, which of the following statements is correct?		
Answer Choices	Responses	
Biosimilars are as effective and safe as the originator drugs	83.33%	195
Biosimilars are as effective but less safe than the originator drugs	3.42%	8
Biosimilars are as safe but less effective than the originator drugs	6.41%	15
Biosimilars are less effective and less safe than the originator drugs	3.85%	9
Do not know	2.99%	7
	**Answered**	**234**
	**Skipped**	**0**
Q8. How confident are you about the use of biosimilars using a scale from 0 (lowest value) to 10 (highest value)?		
	1 (lowest value=	
star	1.28%	3
Q9. How confident are your patients about the use of biosimilars using a scale from 0 (lowest value) to 10 (highest value)?		
	1 (lowest value=	
star	0.85%	2
Q10. Do you think the data on biosimilars extrapolated from other immune mediated inflammatory diseases are also valid in IBD?		
Answer Choices	Responses	
Yes	75.21%	176
No	24.79%	58
	**Answered**	**234**
	**Skipped**	**0**
Q11. Do you think patients should be informed about what a biosimilar is before starting therapy?		
Answer Choices	Responses	
Yes	83.33%	195
No	16.67%	39
	**Answered**	**234**
	**Skipped**	**0**

Q12. Who should provide information to patients about biosimilars?		
Answer Choices	Responses	
Physician (gastroenterologist, internal doctor etc)	86.75%	203
Resident	0.85%	2
Nurse	3.85%	9
Pharmacist	2.56%	6
Government authorities	2.56%	6
Payers/Insurance	1.28%	3
Other (please specify)	2.14%	5
	**Answered**	**234**
	**Skipped**	**0**
Q13. Before prescribing a biological drug, do you explain to patients what a biosimilar drug is and what the originator drug is?		
Answer Choices	Responses	
Yes	70.51%	165
No	29.49%	69
	**Answered**	**234**
	**Skipped**	**0**
Q14. Do you provide patients with data comparing biosimilars and originator drugs?		
Answer Choices	Responses	
Yes	32.91%	77
No	67.09%	157
	**Answered**	**234**
	**Skipped**	**0**
Q15. Do you provide written materials to patients informing them on the use of biosimilars?		
Answer Choices	Responses	
Yes	26.07%	61
No	73.93%	173
	**Answered**	**234**
	**Skipped**	**0**
Q16. Have you ever prescribed biosimilars of infliximab?		
Answer Choices	Responses	
Yes	87.61%	205
No	12.39%	29
	**Answered**	**234**
	**Skipped**	**0**
Q17. Have you ever prescribed biosimilars of adalimumab?		

Answer Choices	Responses	
Yes	83.76%	196
No	16.24%	38
	**Answered**	**234**
	**Skipped**	**0**
Q18. Do you think biosimilars should only be prescribed to naïve patients?		
Answer Choices	Responses	
Yes	19.23%	45
No	72.22%	169
Do not know	8.55%	20
	**Answered**	**234**
	**Skipped**	**0**
Q19. Have you ever switched a patient from the originator drug to the biosimilar?		
Answer Choices	Responses	
Yes	81.62%	191
No	18.38%	43
	**Answered**	**234**
	**Skipped**	**0**
Q20. If you answered yes to question 19, why did you switch from the originator drug to the biosimilar?		
Answer Choices	Responses	
Due to the lower cost of the biosimilar	54.74%	104
Due to allergic reaction to the originator drug	1.05%	2
Due to a loss of response to the originator drug	2.11%	4
Due to unavailability of the originator drug	15.79%	30
Efficacy comparable to the originator drug	3.16%	6
Safety comparable to the originator drug	0.00%	0
Efficacy and safety comparable to the originator drug	10.00%	19
Other (please specify)	13.16%	25
	**Answered**	**190**
	**Skipped**	**44**
Q21. When do you switch from the originator to the biosimilar?		
Answer Choices	Responses	
When the patient achieves clinical remission	5.50%	12
When the patient achieves biochemical remission (normal CRP and/or fecal calprotectin)	1.83%	4
When the patient achieves both clinical remission and biochemical remission	15.14%	33
When the patient achieves endoscopic remission	4.59%	10

Regardless of disease activity	16.97%	37
It is a non-medical decision	55.96%	122
	**Answered**	**218**
	**Skipped**	**16**
Q22. Do you monitor drug trough levels and autoantibodies in patients switched to biosimilars?		
Answer Choices	Responses	
Yes	43.12%	94
No	56.88%	124
	**Answered**	**218**
	**Skipped**	**16**
Q23. Do you have patients who have refused to start therapy with a biosimilar or switch to a biosimilar?		
Answer Choices	Responses	
Yes	46.79%	102
No	53.21%	116
	**Answered**	**218**
	**Skipped**	**16**
Q24. If you have patients who refused to be treated with a biosimilar, what is the proportion of these patients?		
Answer Choices	Responses	
<1%	63.30%	138
<5%	19.27%	42
<10%	7.34%	16
<25%	6.88%	15
<50%	1.83%	4
≥50%	1.38%	3
	**Answered**	**218**
	**Skipped**	**16**
Q25. What is the main reason for patients' refusal of the biosimilar?		
Answer Choices	Responses	
Fear that the drug is not effective	36.24%	79
Fear that the drug is not safe	7.80%	17
Fear that the switch will be made due to a cost issue, neglecting the patient's health	14.22%	31
Fear that the switch may be associated with disease relapse	23.85%	52
Other (please specify)	17.89%	39
	**Answered**	**218**
	**Skipped**	**16**

Q26. Despite the availability of biosimilars, do you prescribe originator drugs?		
Answer Choices	Responses	
Yes	61.01%	133
No	38.99%	85
	**Answered**	**218**
	**Skipped**	**16**
Q27. If you prescribe originator drugs despite the availability of the biosimilars, why do you prefer the originator drug?		
Answer Choices	Responses	
Originator drugs are more effective than biosimilars	7.34%	16
Originator drugs are safer than biosimilars	2.75%	6
Originator drugs are more effective and safer than biosimilars	3.21%	7
I have no biosimilars available in my hospital	11.93%	26
There is little data on biosimilars, I prefer the originator drugs because there is more scientific evidence	17.43%	38
Other (please specify)	57.34%	125
	**Answered**	**218**
	**Skipped**	**16**
Q28. In a patient candidate for biologic therapy, have you ever started IBD therapy using biosimilars?		
Answer Choices	Responses	
Yes	89.91%	196
No	10.09%	22
	**Answered**	**218**
	**Skipped**	**16**
Q29. In a patient who started a biosimilar as first drug, have you ever switched to the originator drug?		
Answer Choices	Responses	
Yes	26.15%	57
No	73.85%	161
	**Answered**	**218**
	**Skipped**	**16**
Q30. If you answered yes to question 29, why were the patients switched from the biosimilar to the originator drug (multiple answers are possible)?		
Answer Choices	Responses	
Due to allergic reaction to the biosimilar	14.81%	8
Due to a loss of response to the biosimilar	27.78%	15
Due to unavailability of the biosimilar	37.04%	20
Other (specify)	3.70%	2
Other (please specify)	16.67%	9
	**Answered**	**54**

	**Skipped**	**180**
Q31. If you answered yes to question 29, did patients switched from the biosimilar to the originator drug achieve/maintain disease remission?		
Answer Choices	Responses	
Yes	83.02%	44
No	16.98%	9
	**Answered**	**53**
	**Skipped**	**181**
Q32. Have you ever switched from one biosimilar to another biosimilar of the same drug (multiple switch)?		
Answer Choices	Responses	
Yes	50.54%	93
No	49.46%	91
	**Answered**	**184**
	**Skipped**	**50**
Q33. If you have multiple switched patients, why were the patients switched from one biosimilar to another (multiple answers are possible)?		
Answer Choices	Responses	
Due to allergic reaction to the first biosimilar	8.15%	15
Due to a loss of response to the first biosimilar	9.24%	17
Due to unavailability of the first biosimilar	44.02%	81
Other (please specify)	44.57%	82
	**Answered**	**184**
	**Skipped**	**50**
Q34. Did multiple switched patients achieve/maintain disease remission?		
Answer Choices	Responses	
Yes	70.11%	129
No	29.89%	55
	**Answered**	**184**
	**Skipped**	**50**
Q35. In a patient treated with the originator drug and then switched to the biosimilar, have you ever prescribed the reverse switch to the originator drug?		
Answer Choices	Responses	
Yes	39.13%	72
No	60.87%	112
	**Answered**	**184**
	**Skipped**	**50**

Q36. If you have patients undergoing reverse switch, what was the reason for reverse switch (multiple answers are possible)?		
Answer Choices	Responses	
Due to allergic reaction to the biosimilar	13.04%	24
Due to a loss of response to the biosimilar	16.30%	30
Due to unavailability of the biosimilar	29.89%	55
Other (please specify)	51.09%	94
	**Answered**	**184**
	**Skipped**	**50**
Q37. Did reverse switched patients achieve/maintain disease remission?		
Answer Choices	Responses	
Yes	69.57%	128
No	30.43%	56
	**Answered**	**184**
	**Skipped**	**50**
Q38. Do you know what the nocebo effect is?		
Answer Choices	Responses	
Yes	86.41%	159
No	13.59%	25
	**Answered**	**184**
	**Skipped**	**50**
Q39. Have your patients ever experienced the nocebo effect?		
Answer Choices	Responses	
Yes	38.59%	71
No	29.35%	54
Do not know	32.07%	59
	**Answered**	**184**
	**Skipped**	**50**
Q40. If your patients experienced the nocebo effect, what is the rate of nocebo effect among your patients?		
Answer Choices	Responses	
None	50.54%	93
<5%	30.98%	57
<10%	12.50%	23
<25%	4.89%	9
<50%	0.54%	1
≥50%	0.54%	1
	**Answered**	**184**

	**Skipped**	**50**
Q41. Do you have patients who underwent a non-medical switch?		
Answer Choices	Responses	
Yes	57.07%	105
No	42.93%	79
	**Answered**	**184**
	**Skipped**	**50**
Q42. Did non-medical switched patients achieve/maintain disease remission?		
Answer Choices	Responses	
Yes	67.39%	124
No	32.61%	60
	**Answered**	**184**
	**Skipped**	**50**
Q43. Does the presence of biosimilars have an impact on your therapeutic choices?		
Answer Choices	Responses	
Yes	73.91%	136
No	26.09%	48
	**Answered**	**184**
	**Skipped**	**50**
Q44. In the near future, will you be prescribing biosimilars of vedolizumab, ustekinumab, and tofacitinib?		
Answer Choices	Responses	
Yes	74.46%	137
No	2.17%	4
Do not know	23.37%	43
	**Answered**	**184**
	**Skipped**	**50**
Q45. Do you think the availability of the biosimilars of vedolizumab, ustekinumab, and tofacitinib will change the treatment algorithm of IBD patients?		
Answer Choices	Responses	
Yes	52.17%	96
No	30.43%	56
Do not know	17.39%	32
	**Answered**	**184**
	**Skipped**	**50**
Q46. How would you implement the use of biosimilars in clinical practice (multiple answers are possible)?		

Answer Choices	Responses	
There is a need to conduct randomized clinical trials to compare the efficacy and safety of biosimilars and originator drugs in patients with IBD	38.59%	71
Long term data are needed	50.54%	93
Non-medical switch	24.46%	45
Patient engagement in the therapeutic decision	32.07%	59
More information should be provided to health care providers	26.63%	49
Government authorities should provide more information to patients about the efficacy and safety of biosimilars	32.61%	60
Patient associations should provide more information on biosimilars	28.80%	53
Other	4.35%	8
	**Answered**	**184**
	**Skipped**	**50**

Supplementary Table 2. CROSS checklist

Section/topic	Item	Item description	Reported on page #
**Title and abstract**			
Title and abstract	1a	State the word "survey" along with a commonly used term in title or abstract to introduce the study's design.	3,4
	1b	Provide an informative summary in the abstract, covering background, objectives, methods, findings/results, interpretation/discussion, and conclusions.	3
**Introduction**			
Background	2	Provide a background about the rationale of study, what has been previously done, and why this survey is needed.	4
Purpose/aim	3	Identify specific purposes, aims, goals, or objectives of the study.	4
**Methods**			
Study design	4	Specify the study design in the methods section with a commonly used term (e.g., cross-sectional or longitudinal).	5,6
	5a	Describe the questionnaire (e.g., number of sections, number of questions, number and names of instruments used).	5,6
Data collection methods	5b	Describe all questionnaire instruments that were used in the survey to measure particular concepts. Report target population, reported validity and reliability information, scoring/classification procedure, and reference links (if any).	5,6

	5c	Provide information on pretesting of the questionnaire, if performed (in the article or in an online supplement). Report the method of pretesting, number of times questionnaire was pre-tested, number and demographics of participants used for pretesting, and the level of similarity of demographics between pre-testing participants and sample population.	5,6
	5d	Questionnaire if possible, should be fully provided (in the article, or as appendices or as an online supplement).	Yes, in Data Suppl.
Sample characteristics	6a	Describe the study population (i.e., background, locations, eligibility criteria for participant inclusion in survey, exclusion criteria).	5
	6b	Describe the sampling techniques used (e.g., single stage or multistage sampling, simple random sampling, stratified sampling, cluster sampling, convenience sampling). Specify the locations of sample participants whenever clustered sampling was applied.	
	6c	Provide information on sample size, along with details of sample size calculation.	NA
	6d	Describe how representative the sample is of the study population (or target population if possible), particularly for population-based surveys.	NA
Survey administration	7a	Provide information on modes of questionnaire administration, including the type and number of contacts, the location where the survey was conducted (e.g., outpatient room or by use of online tools, such as SurveyMonkey).	6
	7b	Provide information of survey's time frame, such as periods of recruitment, exposure, and follow-up days.	6
	7c	Provide information on the entry process:<br>–>For non-web-based surveys, provide approaches to minimize human error in data entry.<br>–>For web-based surveys, provide approaches to prevent "multiple participation" of participants.	6
Study preparation	8	Describe any preparation process before conducting the survey (e.g., interviewers' training process, advertising the survey).	NA
Ethical considerations	9a	Provide information on ethical approval for the survey if obtained, including informed consent, institutional review board [IRB] approval, Helsinki declaration, and good clinical practice [GCP] declaration (as appropriate).	5
	9b	Provide information about survey anonymity and confidentiality and describe what mechanisms were used to protect unauthorized access.	5
Statistical analysis	10a	Describe statistical methods and analytical approach. Report the statistical software that was used for data analysis.	6
	10b	Report any modification of variables used in the analysis, along with reference (if available).	NA

	10c	Report details about how missing data was handled. Include rate of missing items, missing data mechanism (i.e., missing completely at random [MCAR], missing at random [MAR] or missing not at random [MNAR]) and methods used to deal with missing data (e.g., multiple imputation).	5
	10d	State how non-response error was addressed.	NA
	10e	For longitudinal surveys, state how loss to follow-up was addressed.	NA
	10f	Indicate whether any methods such as weighting of items or propensity scores have been used to adjust for non-representativeness of the sample.	NA
	10g	Describe any sensitivity analysis conducted.	NA
**Results**			
Respondent characteristics	11a	Report numbers of individuals at each stage of the study. Consider using a flow diagram, if possible.	8
	11b	Provide reasons for non-participation at each stage, if possible.	NA
	11c	Report response rate, present the definition of response rate or the formula used to calculate response rate.	8
	11d	Provide information to define how unique visitors are determined. Report number of unique visitors along with relevant proportions (e.g., view proportion, participation proportion, completion proportion).	8
Descriptive results	12	Provide characteristics of study participants, as well as information on potential confounders and assessed outcomes.	8
Main findings	13a	Give unadjusted estimates and, if applicable, confounder-adjusted estimates along with 95% confidence intervals and p-values.	NA
	13b	For multivariable analysis, provide information on the model building process, model fit statistics, and model assumptions (as appropriate).	NA
	13c	Provide details about any sensitivity analysis performed. If there are considerable amount of missing data, report sensitivity analyses comparing the results of complete cases with that of the imputed dataset (if possible).	NA
**Discussion**			
Limitations	14	Discuss the limitations of the study, considering sources of potential biases and imprecisions, such as non-representativeness of sample, study design, important uncontrolled confounders.	NA
Interpretations	15	Give a cautious overall interpretation of results, based on potential biases and imprecisions and suggest areas for future research.	16,17
Generalizability	16	Discuss the external validity of the results.	16,17

**Other sections**			
Role of funding source	17	State whether any funding organization has had any roles in the survey's design, implementation, and analysis.	17,18
Conflict of interest	18	Declare any potential conflict of interest.	17,18
Acknowledgements	19	Provide names of organizations/persons that are acknowledged along with their contribution to the research.	17,18

Supplementary Table 3. Preliminary statements

Statements	
1	Biosimilars are as effective and safe as the originator or tolerability issues.
2	Biosimilars can be used both in biologic-naïve patients and in patients already treated with the originator drugs.
3	The main reason for switching from an originator drug to a biosimilar is its lower cost.
4	Switch from an originator drug to a biosimilar can be performed at any time.
5	The switch from an originator drug to a biosimilar is effective and safe.
6	Multiple switches from one biosimilar to another are feasible in case of drug unavailability.
7	There is no sufficient evidence to support multiple switch in case of loss of response to a biosimilar
8	There is no sufficient evidence to support reverse switch to the originator drug
9	There is no need to monitor drug trough levels and autoantibodies in patients switched from the originator drug to the biosimilar
10	The non-medical switch is a way to reduce costs associated with advanced therapies and increase accessibility.
11	In the near future, non-anti-TNF biosimilar drugs are expected to alter the therapeutic algorithm in patients with inflammatory bowel diseases.
